# Supplementary material for: Selective sorting of polymers with different terminal groups using metal-organic frameworks
Source: Nat Commun. 2018 Sep 7;9:3635. doi: 10.1038/s41467-018-06099-z (PMC6128874; doi:10.1038/s41467-018-06099-z)
Supplement: Supplementary file 1 — Supplementary Information [file 41467_2018_6099_MOESM1_ESM.pdf]

Supplementary Information

Selective sorting of polymers with different terminal groups using metal-organic frameworks

Benjamin Le Ouay et al.

Supplementary Methods

Measurements

Powder X-ray diffraction (PXRD) data were collected using a Rigaku SmartLab Diffractometer with Cu Kα radiation. The particle size distribution was obtained by Horiba Partica LA-950 laser diffraction particle size analyzer. Adsorption isotherms of N_2 at 77 K were measured with a Belsorp-mini equipment. Before the adsorption measurements, samples were treated under reduced pressure ($<10^{-2}$ Pa) at 373 K for 5 h. Differential scanning calorimetry (DSC) was conducted with a Seiko Instruments DSC 6220 under an atmosphere of N_2. Scanning electron microscopy (SEM) images were collected using a Hitachi S-3000N SEM system operated at an accelerating voltage of 30 kV. Samples were placed on a conducting carbon tape attached to an SEM grid, and then coated with platinum. Gel permeation chromatograph (GPC) measurements of the PEG was performed in $CHCl_3$ at 40 °C on three linear-type polystyrene gel columns (Shodex K-805L) that were connected to a Jasco PU-980 precision pump, a Jasco RI-930 refractive index detector, and a Jasco UV-970 UV–vis detector set at 256 nm. ^{1}H NMR spectra were obtained using a JEOL A-500 spectrometer operating at 500 MHz. Solid-state NMR measurement was performed on a 9.4 T Bruker solid-state NMR instrument with an Advance III 400 MHz spectrometer and a double resonance 4 mm magic angle spinning probe. ^{1}H-^{13}C heteronuclear correlation (HETCOR) with frequency-switched Lee–Goldburg (FSLG) homonuclear decoupling was conducted. The HETCOR spectrum was obtained using a recycle delay of 2 s with a spinning rate of 10 kHz. FSLG contact time of ^{1}H-^{13}C cross-polarization was 2 ms. A single crystal of **2** containing H−PEG−H (0.6 kDa) was mounted using MiTeGen's MicroMount™. Intensity data were collected at 103 K in flowing low temperature nitrogen gas on a Rigaku XtaLAB P200 with VariMax Mo Optic with MoKα radiation (λ = 0.71075 Å) and a confocal monochromator. The structure was solved by direct methods and refined by full-matrix least-squares cycles in SHELX2018/1.[1,2] All non-hydrogen atoms were refined with anisotropic thermal parameters. Hydrogen atoms attached to C were located at geometrically calculated positions and refined with isotropic thermal parameters. The guest PEG molecules included in the channels showed severe disorder and the *SQUEEZE* command in *PLATON*[3,4] was used in the structure refinement.

MD simulation

All the molecular dynamics (MD) simulations were executed using the pmemd module of the Amber12 package, which was modified to handle covalent bonds across the periodic boundary. All the density functional theory (DFT) calculations were executed at the level of M06-2X/6-31+G(d) using the Gaussian09 package. The guest PEG molecules are modeled as X–(O-CH_2-CH_2)$_{14}$–O–X (terminal groups X are H atom, Tr group, or Me group) by general Amber force field (GAFF) and their atomic charges are assigned by the Merz–Kollman scheme. We use previously reported force field parameters for the MOF framework model of **1b**.[5] For the **1a** model, we use the same parameters for the Zn dimers and triethylenediamine in the model of **1b** and newly prepare those for the 1,4-naphthalenedicarboxylate. By using DFT calculation results of Li^+ capped 1,4-naphthalenedicarboxylate neutral model, the atomic charges are assigned by the Merz–Kollman scheme and intramolecular parameters (bond, angle, and dihedral terms) are determined to reproduce the rotational barrier of the naphthalene group rotation.

The MD simulation models are composed of the host MOF framework (**1a** or **1b**) and guest PEG molecules with different terminal groups (H–PEG–H, Tr–PEG–Tr, or Me–PEG–Tr) under periodic boundary conditions. We executed the simulation of four systems, with the combinations that follow: (1) **1a** with H–PEG–H, (2) **1a** with Tr–PEG–Tr, (3) **1a** with Me–PEG–Tr, and (4) **1b** with Tr–PEG–Tr. The MOF framework models are built by aligning the unit cell 4 × 4 × 10 with the unit cell size 10.921 × 10.921 × 9.611 Å^3 (**1a**) or 10.948 × 10.948 × 9.804 Å^3 (**1b**) along the *a*-, *b*-, and *c*-axis direction. The MOF framework models are periodically connected along the *a*- and *b*-axes and each of the terminal Zn atom on the [001] surface are capped by a CH_3 group.

We executed MD simulations with the following simulation parameters. The integration time step is 1 ps and the SHAKE algorithm is applied to keep the distances of bonds involving hydrogen atoms. The temperature is controlled to be at 373 K by the weak-coupling algorithm under constant temperature and volume ensemble.

The initial structures in the MD simulation systems were created by placing 24 PEG chains on one side of the [001] surface of the MOF framework with the periodic boundary box size 43.684 × 43.684 × 200.0 Å^3 (**1a**) or 43.792 × 43.792 × 200.0 Å^3 (**1b**). Then, the PEG molecules on the MOF framework surface were equilibrated by a 300 ps MD simulation with the constraint of the atomic positions of the MOF framework by belly algorithm. Finally, production MD simulations were executed for 500 ns.

Synthesis of MOFs

Synthesis of **1a**: **1a** was prepared according to literature.[6] 3.36 mmol of $Zn(NO_3)_2$•$6H_2O$, 3.36 mmol of 1,4-naphthalenedicarboxylic acid, 1,67 mmol of triethylenediamine were dissolved in 20 ml of DMF, then heated at 120 °C for 48 h in a steel autoclave. After the reaction, the resulting white powder was collected by centrifugation and washed several times with dehydrated DMF. The activated MOF **1a** was then obtained by evacuating the solvent at 140 °C under reduced pressure, and stored over desiccating silica gel.

Synthesis of **1b**: **1b** was prepared according to literature.[7] 3.36 mmol of $Zn(NO_3)_2$•$6H_2O$, 3.36 mmol of 1,4-benzenedicarboxylic acid, 1.67 mmol of triethylenediamine were dissolved in 20 ml of DMF, then heated at 120 °C for 48 h in a steel autoclave. After reaction, the resulting white powder was collected by centrifugation and washed several times with dehydrated DMF. The activated MOF **1b** was then obtained by evacuating the solvent at 140 °C under reduced pressure, and stored over desiccating silica gel.

Synthesis of **2**: **2** was prepared according to the literature.[8] 3.4 mmol of $Co(NO_3)_2$•$6H_2O$, 3.4 mmol of 2,6-naphthalenedicarboxylic acid and 1.7 mmol of 4,4′-bipyridine were dissolved in 600 ml DMF and heated at 120 °C for 24 h. After cooling down, the resulting powder was collected by filtration, and washed with dehydrated DMF and methanol to yield **2**•MeOH as a green powder. MeOH was then evacuated at room temperature under reduced pressure (300 Pa then 3 Pa) to yield **2** without guest (purple powder). The evacuated **2** was stored at 4 °C over desiccating silica gel.

Synthesis of functionalized PEG

Note that in this article, the molecular weight indicated for functionalized PEGs refers to the molecular weight of the parent hydroxylated chain.

Synthesis of tritylated PEGs: Tr–PEG–Tr (2 kDa and 20 kDa) and Me–PEG–Tr (2 kDa) were prepared by coupling the corresponding hydroxylated PEGs with trityl chloride.[9] In a typical synthesis, PEG (ca. 5 g) was dissolved in anhydrous dichloromethane. Excess triethylamine and trityl chloride (2.5 equivalents per OH group in the case of PEG (2 kDa), and 10 equivalents in the case of PEG (20 kDa)) were added. Reactions were conducted in closed vials for 96 h at room temperature. After reaction, the organic phase was washed with aqueous NH_4Cl and deionized water, dried over $MgSO_4$, then evaporated under reduced pressure. Tritylated PEG was further purified by dissolution in a dichloromethane/diethyl ether mixture (1:99 vv) followed by evacuation at 300 kPa to cause the precipitation of PEG. The conversion was quantitative, as determined by comparison of the integrals of ^{1}H NMR peaks for Tr groups and main chain.

Synthesis of alkylated PEGs: Me–PEG–Me, Et–PEG–Et and Bu–PEG–Bu were prepared by Williamson ether synthesis from the corresponding hydroxylated PEGs.[10] In a typical synthesis, H–PEG–H (ca. 5 g) was dissolved in 100 ml anhydrous toluene, and maintained at 0 °C. Excess NaH (suspension in oil) was then added and left to react for 15 min. After this, the desired alkyl halide (MeI, EtBr, or *n*-BuBr, 5 equivalents per OH group) was added. The reaction temperature was then maintained at 0 °C for 1 h then progressively increased to 60 °C for 24 h. Afterwards, the reaction temperature was decreased to 0 °C and MeOH was added to neutralize the remaining NaH. The organic phase was then washed with aqueous NH_4Cl and deionized water, then evaporated under

reduced pressure. The resulting mixture was dissolved in water, then washed with diethyl ether and extracted with dichloromethane. The dichloromethane phase was then filtered over activated charcoal and celite, and evaporated under reduced pressure at 60 °C to yield the alkylated PEG. The conversion was quantitative, as determined by comparison of the integrals of ^{1}H-NMR peaks for alkyl groups and main chain.

DSC analysis to determine the enthalpy of PEG insertion

Guest-free **1a** and solid PEG were placed together in a DSC crucible. A very slow heating ramp (1 °C min^{-1}) was used to allow for the quantitative insertion of PEG during the first DSC cycle. Upon heating, the sample exhibited an endothermic peak corresponding to PEG melting, followed by an exothermic peak originating from PEG insertion. By contrast, neat PEG exhibited only the endothermic peak. The integral of the exothermic peak corresponds the heat released upon introduction of PEG. Because the two peaks cannot be integrated separately, the total integral is compared to that of melting PEG without MOF. The released heat can be determined by difference.

DSC analysis to determine the kinetics of PEG insertion

A known amount of MOF (m_{MOF}) and PEG (m_{PEG}) was introduced into a DSC crucible. The amount of PEG added was slightly above the maximal capacity of the MOF, to allow for a complete introduction. A very short heating treatment was performed to guarantee the homogeneity of PEG and MOF. The sample was then submitted to successive heating plateaus to allow some insertion to proceed (**1a**: 70 °C for 10 min, **2**: 45 °C for 10 min). Between each plateau, the sample was cooled down to 10 °C, then heated again, so a melting of PEG could be observed after each plateau. The enthalpy of fusion (*H(N*th *plateau)*) corresponds to free PEG remaining outside of the MOF, and the quantity of PEG inserted after each plateau could be deduced by difference. The loading L, expressed in w% of the MOF (without guest), was determined using the formula $L(N) = \frac{H_0 - H(N)}{H_0} \frac{m_{\mathrm{PEG}}}{m_{\mathrm{MOF}}}$, where H_0 is the melting enthalpy of the corresponding PEG measured in absence of MOF.

Separation of Me–PEG–Me and H–PEG–H using **2**

The selectivity of insertion of Me–PEG–Me and H–PEG–H into **2** was determined from the ^{1}H-NMR spectra of etched composites in DMSO-d_6/DCl (37 w%in D_2O) (see Supplementary Fig. 22 and Supplementary Table 2). Me–PEG–Me possess two characteristic peaks: a sharp singlet (H_a, δ= 3.15 ppm) corresponding to terminal Me groups, and a massif between 3.20 and 3.65 ppm (H_b), corresponding to the main chain ethylene glycol (EG). The integral of the Me peak is always normalized to 6 in this study. In a mixture, only Me–PEG–Me contributes to the peak of the Me groups, while both Me–PEG–Me and H–PEG–H contribute to the integral of the EG massif I_{EG}. The molar fraction of Me–PEG–Me $x_{\mathrm{Me\text{-}PEG\text{-}Me}}$ was deduced from the equation $x_{\mathrm{Me\text{-}PEG\text{-}Me}} = I_{\mathrm{EG,Me\text{-}PEG\text{-}Me}}/I_{\mathrm{EG}}$. In addition, peaks of the MOF ligands were observed in the aromatic region, and could be used to determine the PEG loading L in **2** (expressed in wt% of the mass of empty MOF). Considering I_{NDC} the integral for protons in position 3 and 7 of 2,6-naphthalenedicarboxylic acid (H_c, doublet, δ = 8.16 ppm), L could be calculated using the formula: $L = \frac{I_{\mathrm{EG}}}{I_{\mathrm{NDC}}} \frac{M_{\mathrm{EG}}}{4} \frac{2}{M_2} = 0.0627 \frac{I_{\mathrm{EG}}}{I_{\mathrm{NDC}}}$, with M_{EG} = 44.05 g mol^{-1} for 4 integrated protons, and M_2 = 351.20 g mol^{-1} for 2 integrated protons.

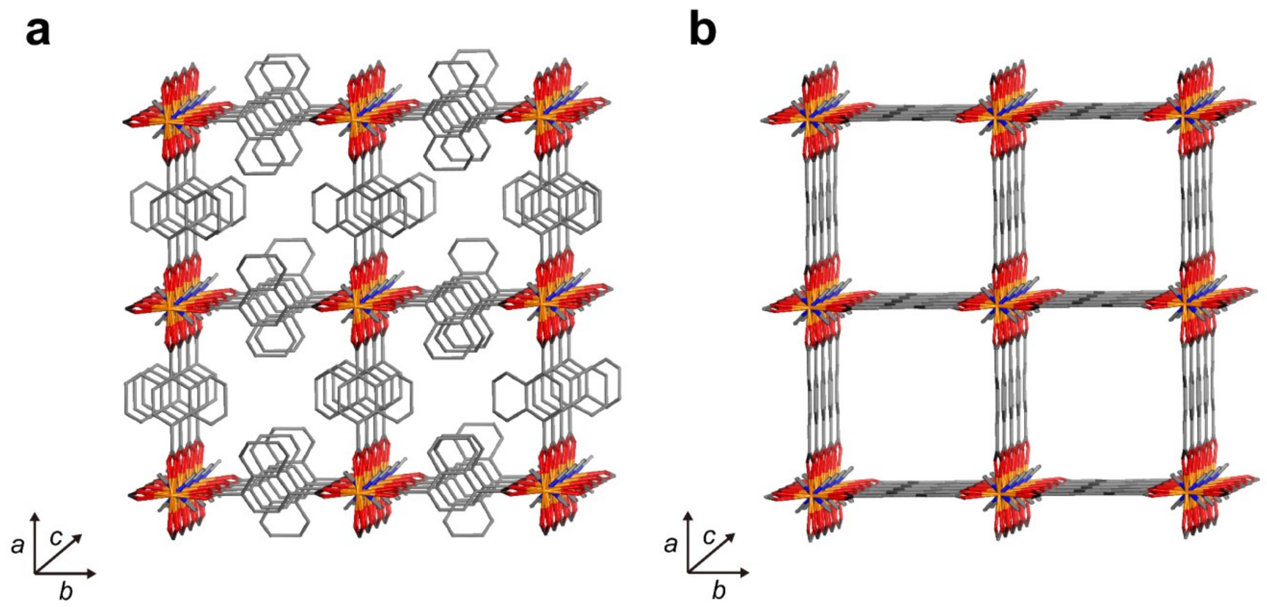

Supplementary Figure 1. Crystal structures of MOFs. (**a**) **1a** and (**b**) **1b** (Zn, orange; O, red; N, blue; C, gray). Hydrogen atoms are omitted for clarity.

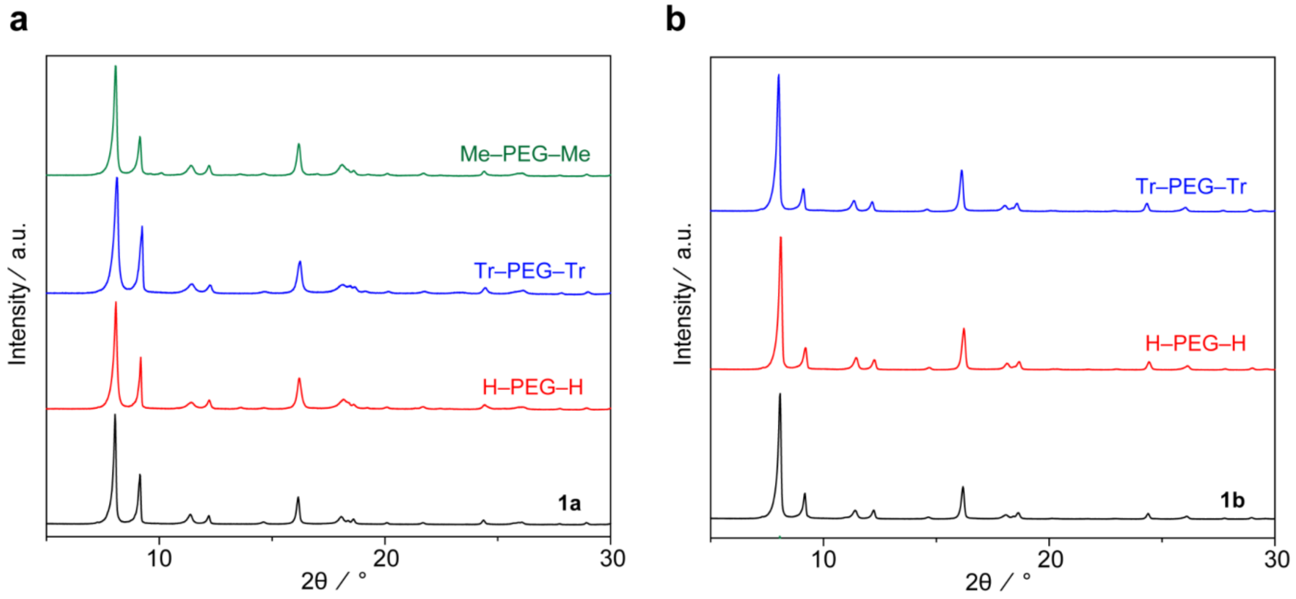

Supplementary Figure 2. PXRD patterns of MOFs with PEG. (**a**) PXRD patterns of **1a** and **1a** after treating with H–PEG–H, Tr–PEG–Tr, and Me–PEG–Me. (**b**) PXRD patterns of **1b** and **1b** after treating with H–PEG–H and Tr–PEG–Tr. M_n of PEG was 2 kDa. All the diagrams show a maintenance of crystallinity for **1**.

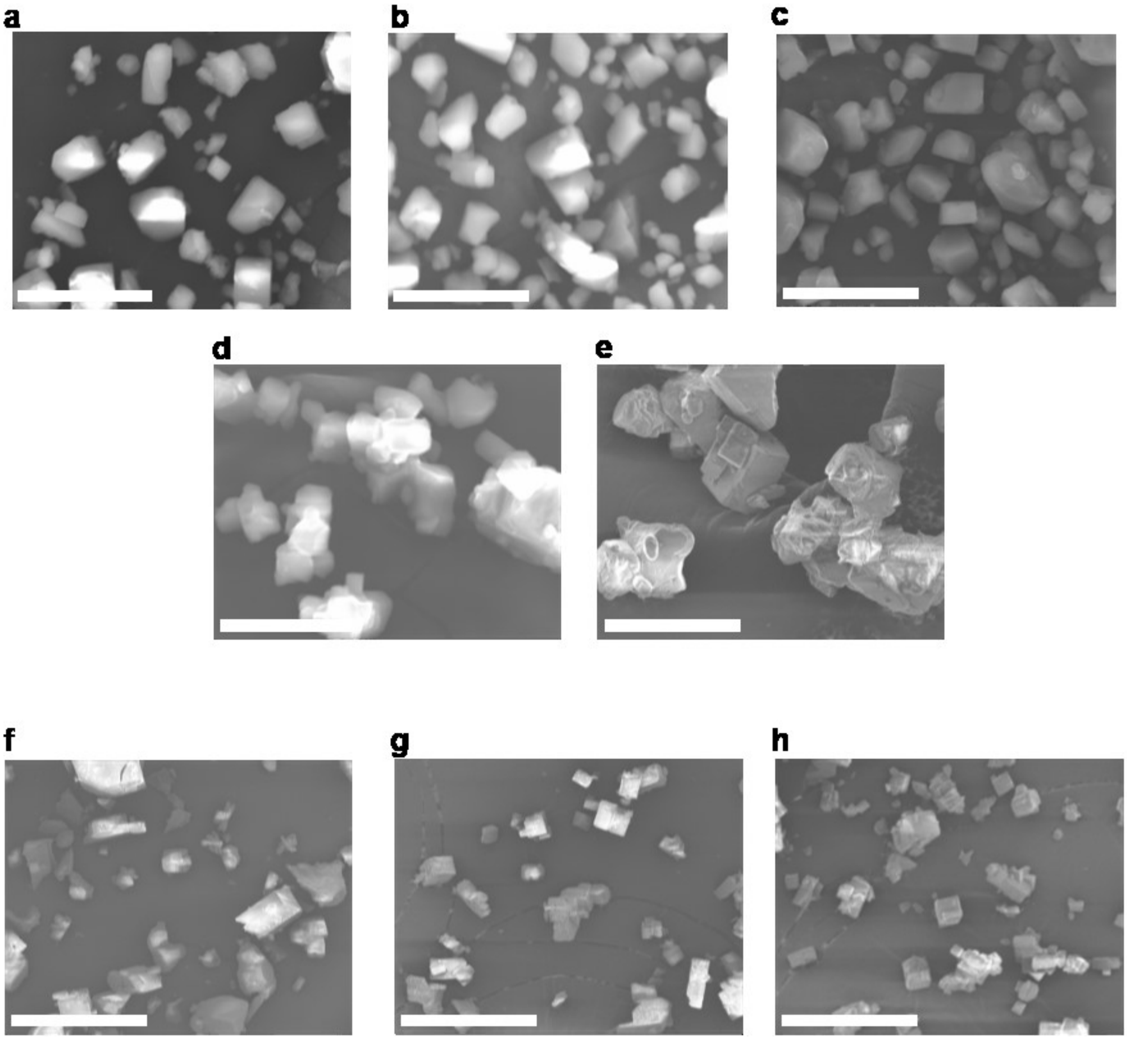

Supplementary Figure 3. SEM images of **1** before and after the treatment with PEG. (**a**) Pristine **1a**. (**b**–**e**) **1a** after treatment with (**b**) H–PEG–H, (**c**) Me–PEG–Me, (**d**) Tr–PEG–Tr, (**e**) Me–PEG–Tr. (**f**) Pristine **1b**. (**g–h**) **1b** after treating with (**g**) H–PEG–H and (**h**) Tr–PEG–Tr. All scale-bars represent 50 μm. M_n of PEG was 2 kDa. The crystal size and morphology of **1a** did not change during the incorporation of H–PEG–H and Me–PEG–Me, indicating the full accommodation of the PEG chains in the nanochannels. In contrast, the SEM image of **1a** and Tr–PEG–Tr composite exhibits the agglomeration of MOF particles, possibly stuck together by Tr–PEG–Tr outside the **1a**. Selective exclusion of Tr–PEG–Tr chains from **1a** is caused by the steric hindrance of the bulky terminal group. In contrast, maintenance of the morphology of **1b** after the treatment with Tr–PEG–Tr suggested that **1b** enabled the encapsulation of Tr–PEG–Tr chains because of pore size slightly larger than Tr groups.

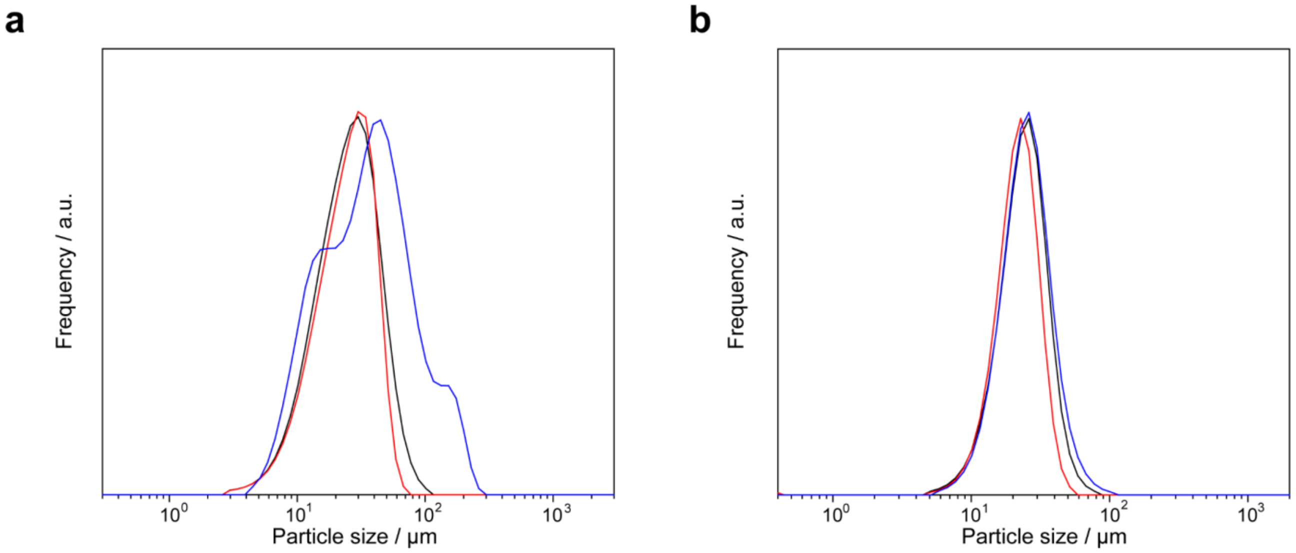

Supplementary Figure 4. Particle size distributions of **1** before and after the addition of PEG evaluated by laser light scattering. (**a**) **1a** before (black) and after the addition of H–PEG–H (red) and Tr–PEG–Tr (blue). (**b**) **1b** before (black) and after the addition of H–PEG–H (red) and Tr–PEG–Tr (blue). M_n of PEG was 2 kDa. When H–PEG–H was inserted into **1**, both **1a** and **1b** presented the particle size distributions similar to that of the pristine MOF, suggesting that H–PEG–H was completely included. In contrast, the composite of **1a** and Tr–PEG–Tr presented several modes in its size distribution, as the polymer remaining outside the MOF caused the aggregation of particles. These additional modes were not observed for **1b**, indicating that insertion of Tr–PEG–Tr was possible in this MOF. These results are consistent with the SEM observations (Supplementary Fig. 3).

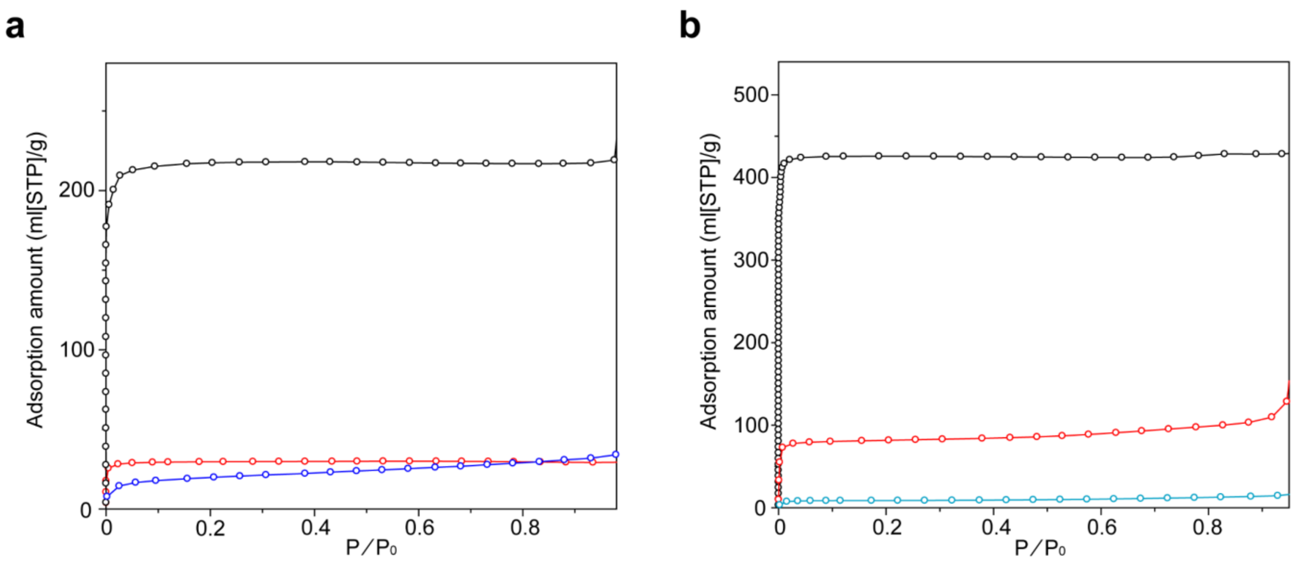

Supplementary Figure 5. N_2 adsorption isotherms at 77 K of **1** before and after treating with PEG. (**a**) **1a** (black) and **1a** after treating with H–PEG–H (2 kDa) (red) and H–PEG–H (20 kDa) (blue). The adsorption isotherms of **1a** including H–PEG–H showed a drastic decrease in adsorption capacity compared with that of pristine host **1a**, indicating the presence of PEG chains within the nanopores. (**b**) **1b** (black) and **1b** after treating with H–PEG–H (2 kDa) (red) and Tr–PEG–Tr (2 kDa) (turquoise). A decrease in the adsorption amount confirmed that **1b** can allow for the encapsulation of both Tr–PEG–Tr and H–PEG–H owing to a pore size larger than the Tr group.

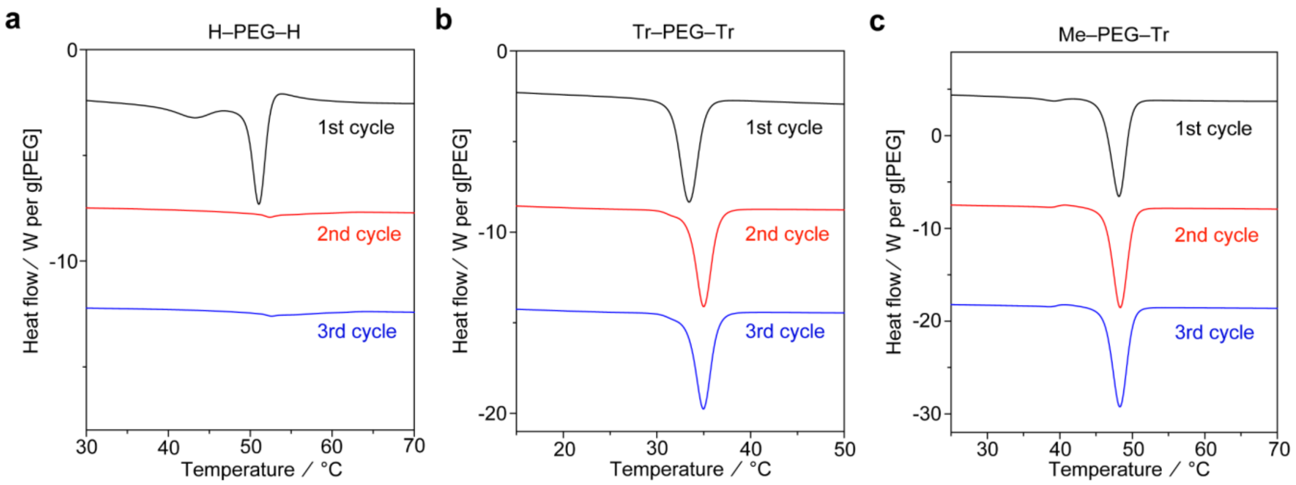

Supplementary Figure 6. DSC heating curves of **1a** with PEG before the heating treatment. (**a**) H–PEG–H, (**b**) Tr–PEG–Tr, and (**c**) Me–PEG–Tr. M_n of PEG was 2 kDa. Scan rate: 10 °C min^{-1} ; range: 0 °C to 100 °C. Samples were analyzed after evacuation of the solvent, but before thermal annealing. For H–PEG–H, the first cycle presents a peak with a lower intensity than that of neat H–PEG–H, and an early prepeak. This was attributed to the partial insertion (ca. 30%) of PEG in the MOF that occurred during the solvent evacuation at room temperature. The PEG melting peak disappears during the second cycle and onward. This was because of the DSC analysis cycle that can be seen as a thermal annealing process. For Tr–PEG–Tr and Me–PEG–Tr, the presence of bulky terminal groups prevents the insertion, even at high temperature. The melting peak of PEG remained unchanged on all cycles during the analysis.

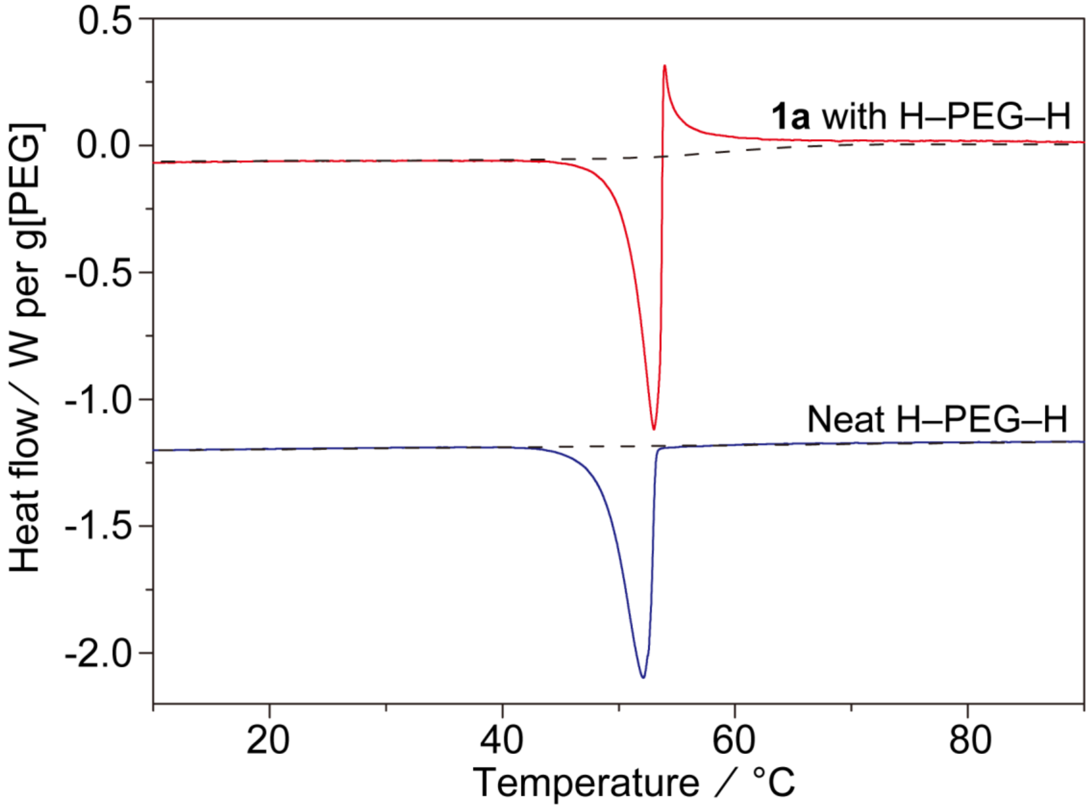

Supplementary Figure 7. DSC heating curves of neat H–PEG–H (blue) and **1a** with H–PEG–H (red) during an insertion experiment. The dotted line corresponds to the baseline used for integration. Scan rate: 1 °C min^{-1}. M_n of PEG used in these experiments was 2 kDa. For the introduction of H–PEG–H in **1a**, the heat of adsorption was determined as 175 J per g of PEG inserted, corresponding to 7.7 kJ per mol of the repeating unit in PEG. This value is comparable to that obtained from MD simulation for adsorption of oligo(ethylene glycol) (14mer) in a MOF.[5]

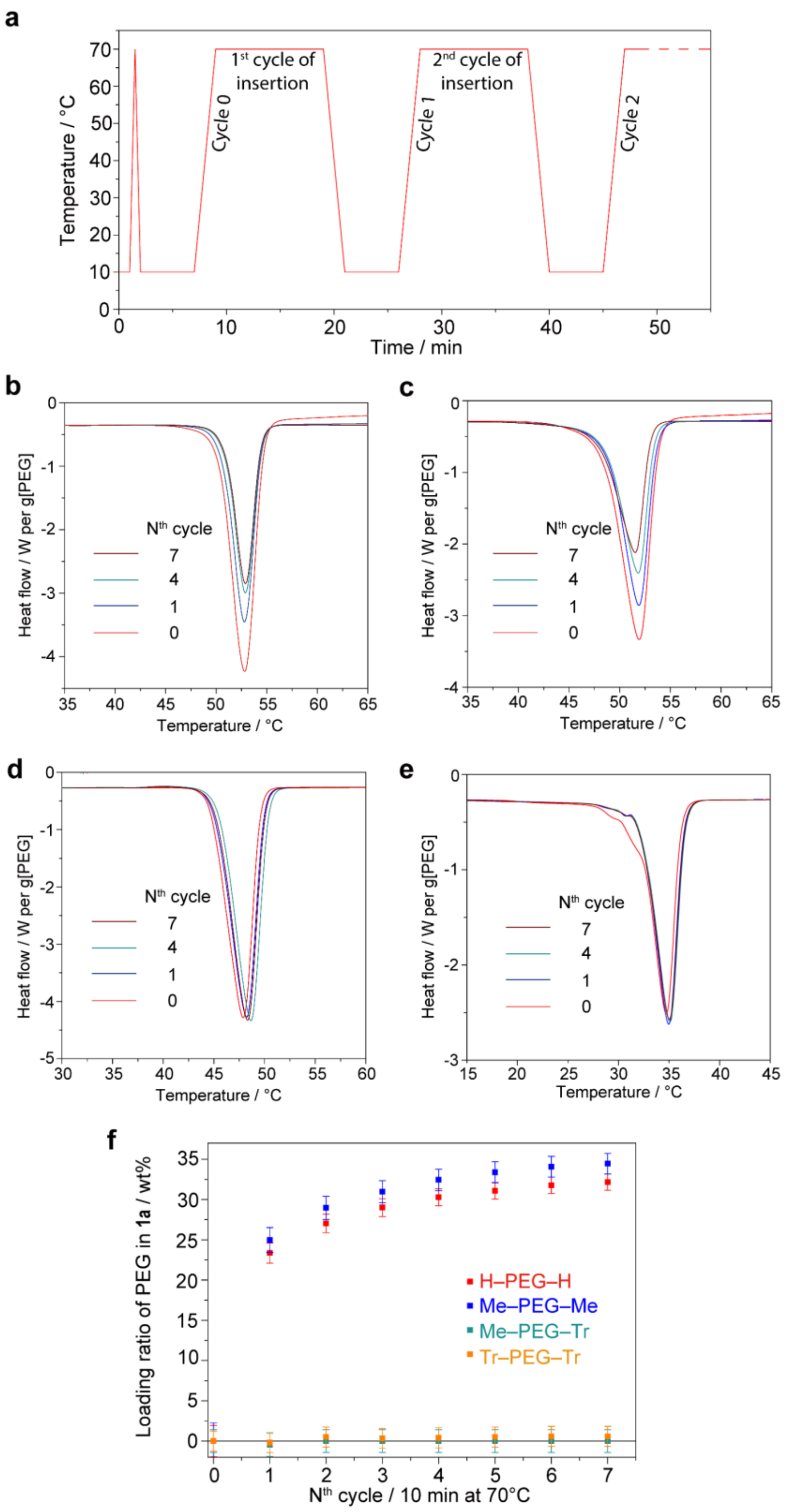

Supplementary Figure 8. DSC determination of the insertion kinetics of neat PEG in **1a**. (**a**) Temperature program used for the monitoring of insertion. (**b**–**e**) DSC curves of H–PEG–H (**b**), Me–PEG–Me (**c**), Me–PEG–Tr (**d**), and Tr–PEG–Tr (**e**) in contact with **1a** heating rate: 5 °C min^{-1}). (**f**) Insertion kinetics of neat PEG (2 kDa) in **1a**, as determined by DSC (error bars: s.d. of the propagated measurement uncertainties). This analysis indicates a progressive insertion of H–PEG–H and Me–PEG–Me in **1a**, until the maximal loading is reached. These two PEGs showed very small differences in their insertion kinetics in **1a**. Meanwhile, tritylated PEGs showed no insertion because of their bulky terminal groups.

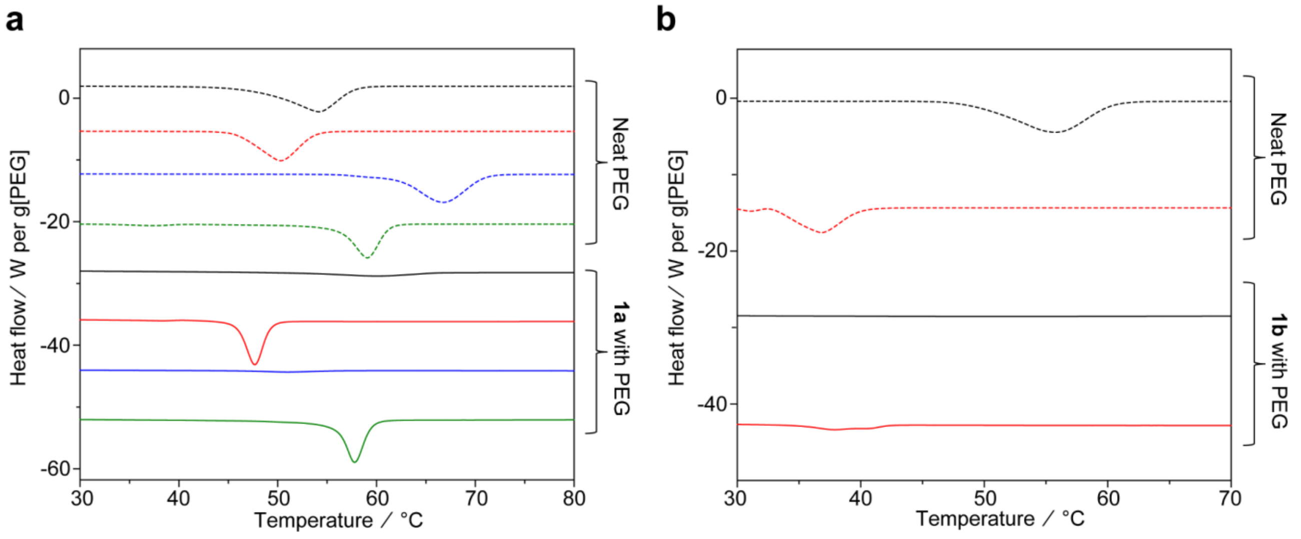

Supplementary Figure 9. DSC heating curves of neat PEG (dotted lines) and **1** with PEG after thermal annealing (solid lines). (**a**) **1a** with Me–PEG–Me (2 kDa) (black), Me–PEG–Tr (2 kDa) (red), H–PEG–H (20 kDa) (blue), and Tr–PEG–Tr (20 kDa) (green). (**b**) **1b** with H–PEG–H (2 kDa) (black) and Tr–PEG–Tr (2 kDa) (red). Scan rate: 10 °C min^{-1}. For **1a**, the composites do not present a melting peak for H–PEG–H and Me–PEG–Me in the bulk state, indicating a complete introduction of these PEGs. The endothermic peak of neat Tr–PEG–Tr was still observed in the DSC profile of **1a** with Tr–PEG–Tr. This is in sharp contrast with the case of **1b** that allowed for an introduction of both H–PEG–H and Tr–PEG–Tr owing to the large pore size than Tr group.

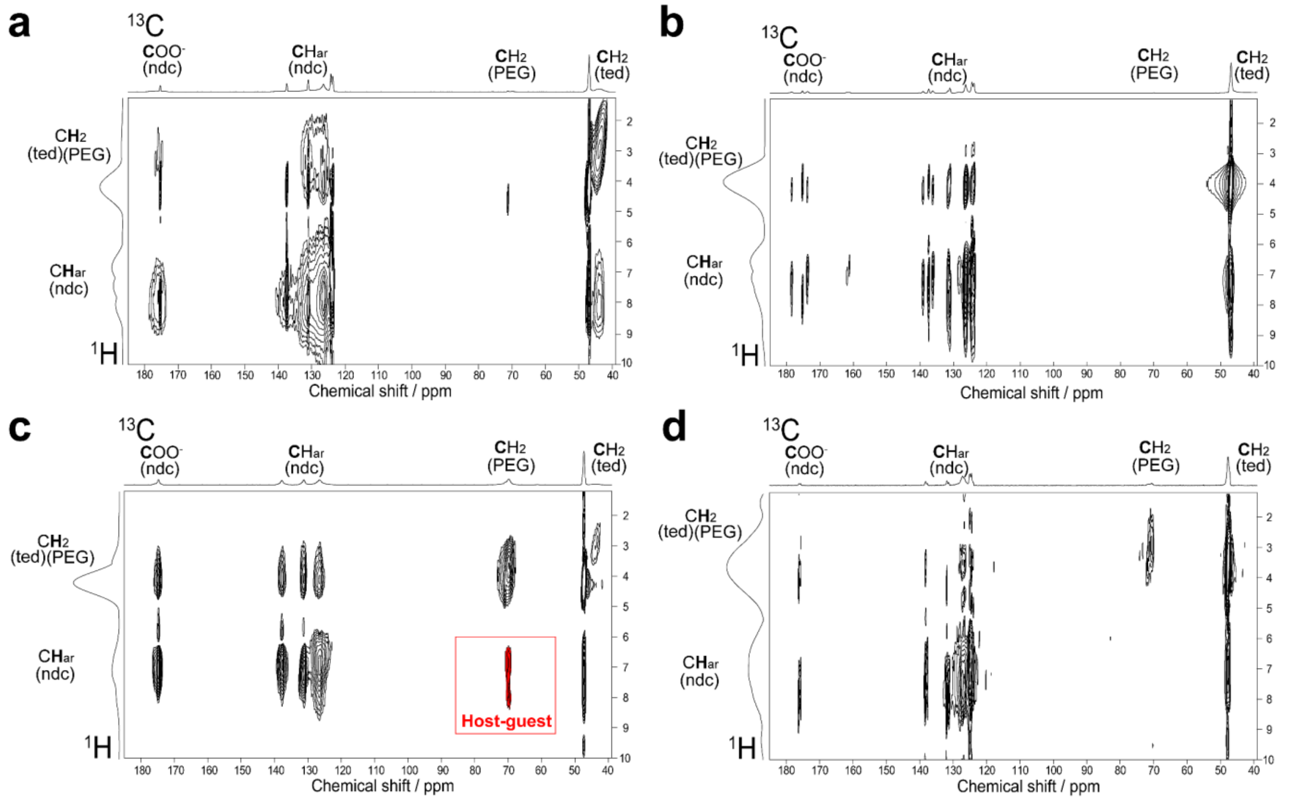

Supplementary Figure 10. Solid-state NMR spectra of **1a** with PEGs. 2-D ^{1}H-^{13}C HETCOR NMR spectra of **1a** with (**a**) Tr–PEG–Tr (2 kDa) (**b**) Me–PEG–Tr (2 kDa), (**c**) H–PEG–H (20 kDa), and (**d**) Tr–PEG–Tr (20 kDa). 2-D ^{1}H–^{13}C HETCOR NMR spectra of **1a** including H–PEG–H with a molecular weight of 20 kDa exhibited a cross-peak associated with the intermolecular host–guest interactions that occurred through dipole–dipole interactions at a short distance of less than 5 Å. This is a clear indication that H–PEG–H was accommodated in the nanochannels of **1a**, which is in sharp contrast with Tr–PEG–Tr. These results indicated that **1a** could selectively encapsulate H–PEG–H in the nanochannels.

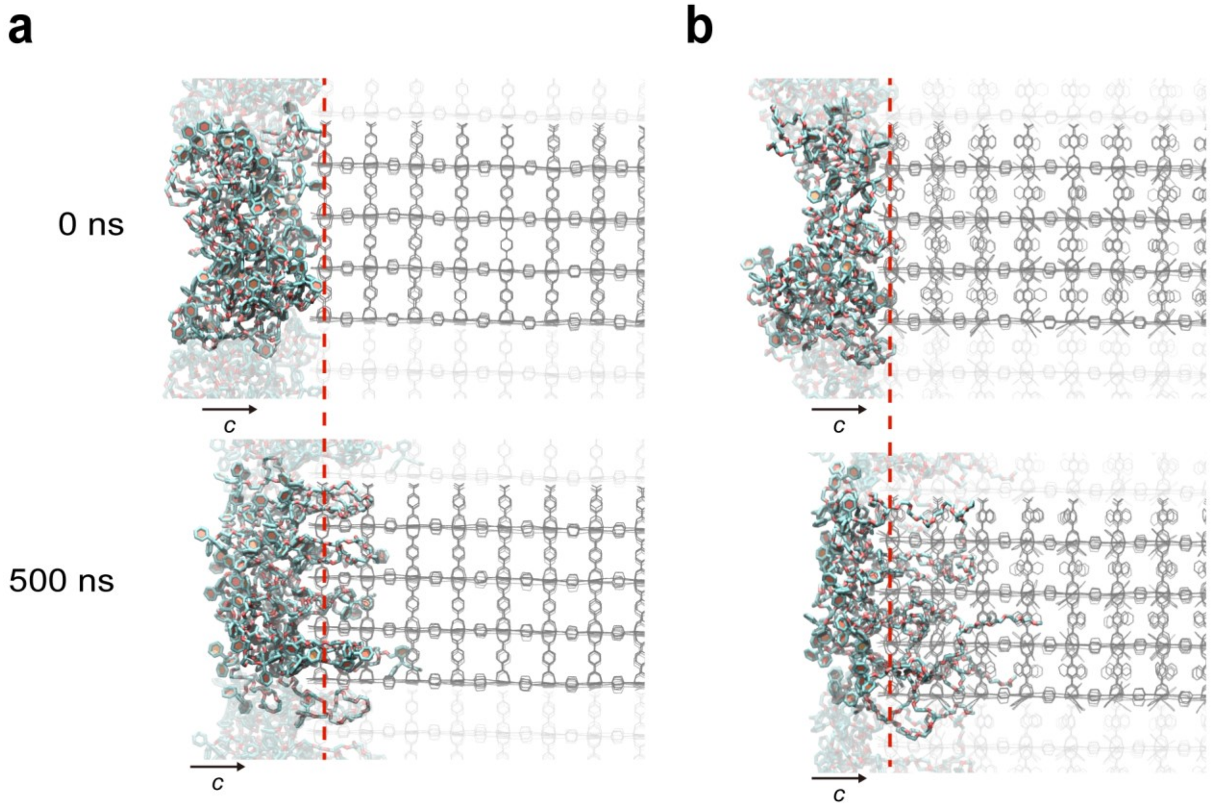

Supplementary Figure 11. MD simulation of PEG permeation into MOFs. (**a**) MD simulation snapshots of permeation of Tr–PEG–Tr into the framework of **1b** from the [001] surface at 373 K. In the main manuscript, we show that the selective exclusion of Tr–PEG–Tr from the nanochannels of **1a** was attributed to the steric hindrance of terminal groups as well as the π–π interaction between Tr groups and the ligands. In contrast, Tr–PEG–Tr can spontaneously permeate the nanochannels of **1b** from [001] surface. These results demonstrate that precise tuning of pore size at the molecular level is of key importance for discriminating terminal groups of polymer chains based on steric hindrance. (**b**) MD simulation snapshots of permeation of Me–PEG–Tr into the framework of **1a** from the [001] surface at 373 K. The MD simulation showed that the Me-terminus of PEG entered the channels of **1a**; however, the adsorption of Me–PEG–Tr remained limited only to the surface of MOF crystals, as the Tr-terminus remained excluded by the pore windows.

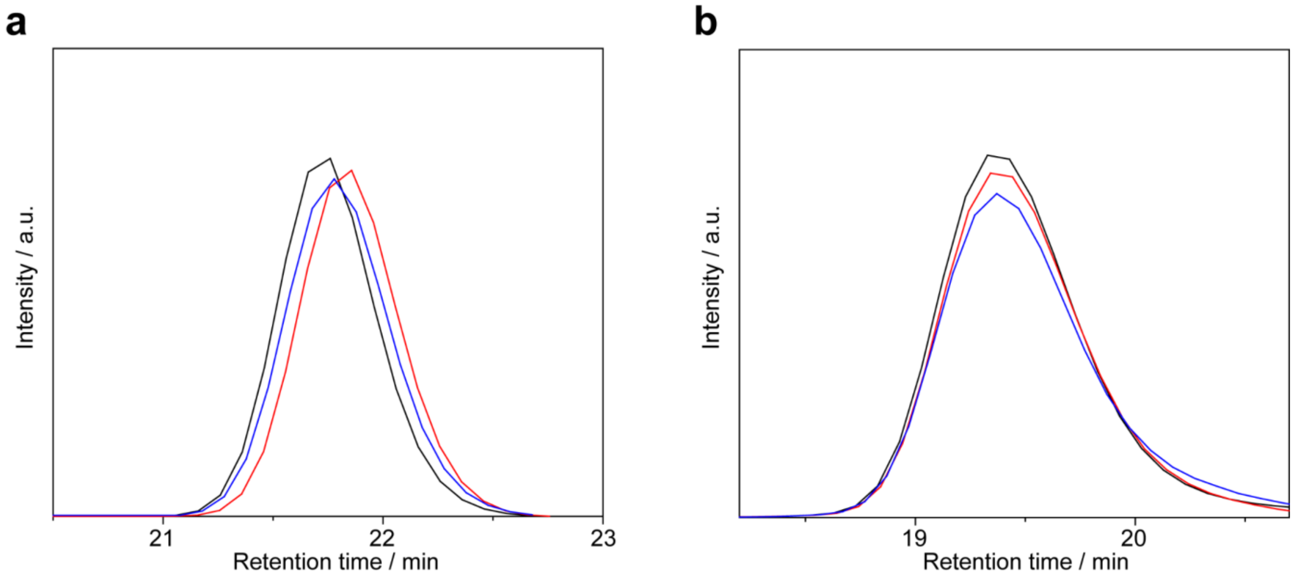

Supplementary Figure 12. GPC profiles of H–PEG–H (black), Tr–PEG–Tr (red), and a mixture of H–PEG–H and Tr–PEG–Tr (blue). (**a**) PEG (2 kDa) and (**b**) PEG (20 kDa). Tritylated PEGs are almost undistinguishable from the pristine H–PEG–H with the same chain length using this technique. Their separation cannot be accomplished by GPC.

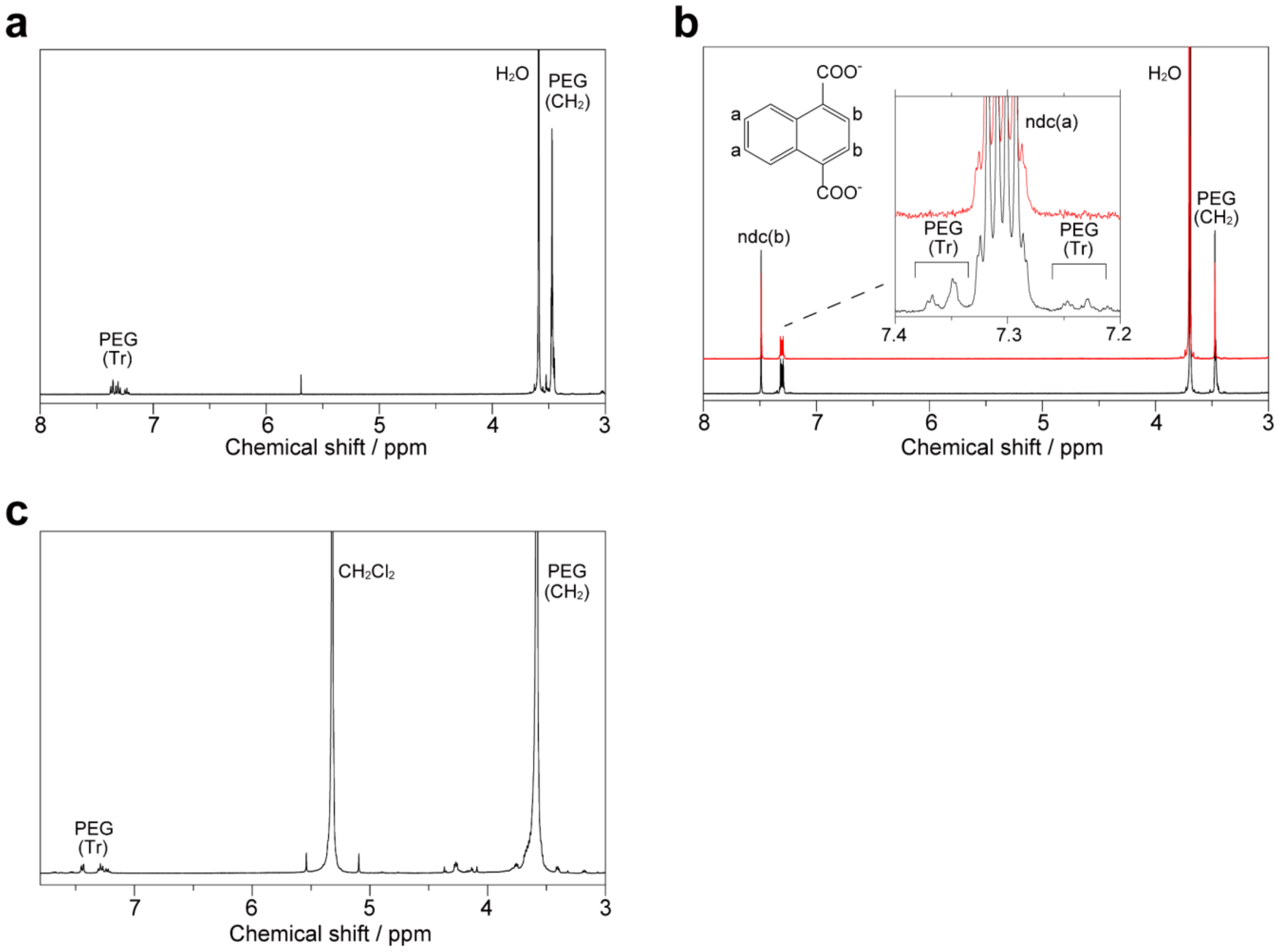

Supplementary Figure 13. ^{1}H-NMR analysis of the separation of H–PEG–H and Tr–PEG–Tr by **1a**. (**a**) ^{1}H-NMR spectrum of free PEG collected by washing treatment. (**b**) ^{1}H-NMR spectra of composites before (black) and after (red) washing treatment, measured in a digesting solution (D_2O/DMSO-d_6/Na_4EDTA). Washing treatment enabled the complete removal of Tr–PEG–Tr outside **1a**, as evidenced by the loss of the peak between 7.2 to 7.4 ppm corresponding to the Tr group. (**c**) ^{1}H-NMR spectrum of free PEG (20 kDa) collected by washing treatment in CD_2Cl_2. The integral ratio between trityl and methylene groups of the obtained PEG was identical to the pristine Tr–PEG–Tr, indicating analytical purity. These results clearly demonstrate that this method is very simple yet can achieve the excellent purification efficiency of mixtures of H–PEG–H and Tr–PEG–Tr with only a marginal difference at the terminal groups. For the separation systems of PEG 2 kDa and 20 kDa, the initial ratios of H –PEG–H and Tr–PEG–Tr mixtures were 5:5 and 2:8, respectively.

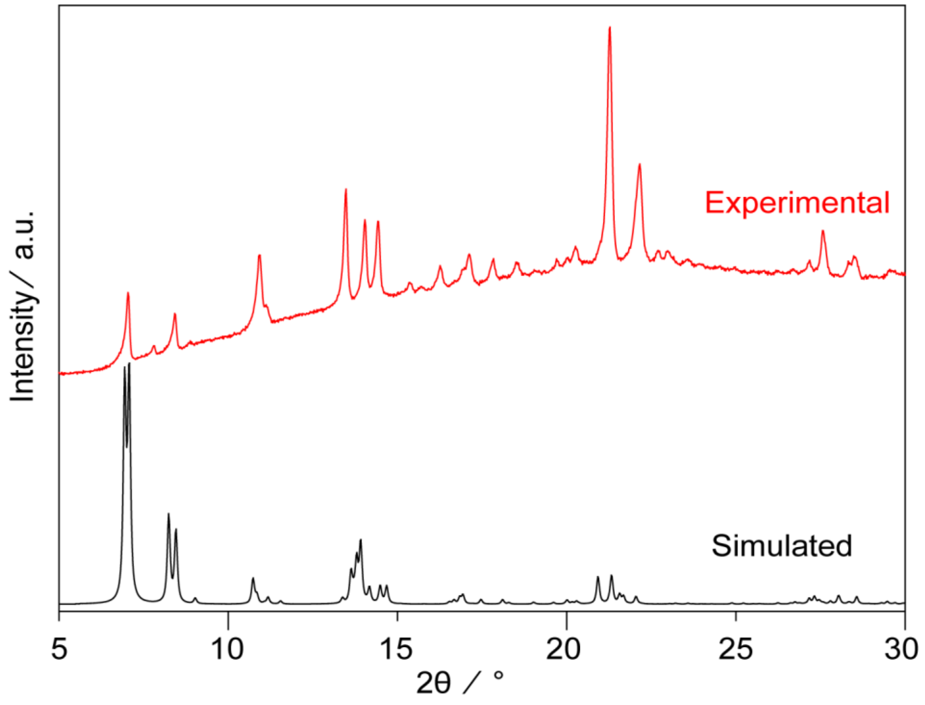

Supplementary Figure 14. PXRD patterns of **2** including H–PEG–H obtained by the solvent-evacuation method, compared with a pattern simulated from the crystal structure. M_n of PEG used in these experiments was 0.6 kDa. The differences in peak intensities originate from the absence of guest in the crystal structure reported.

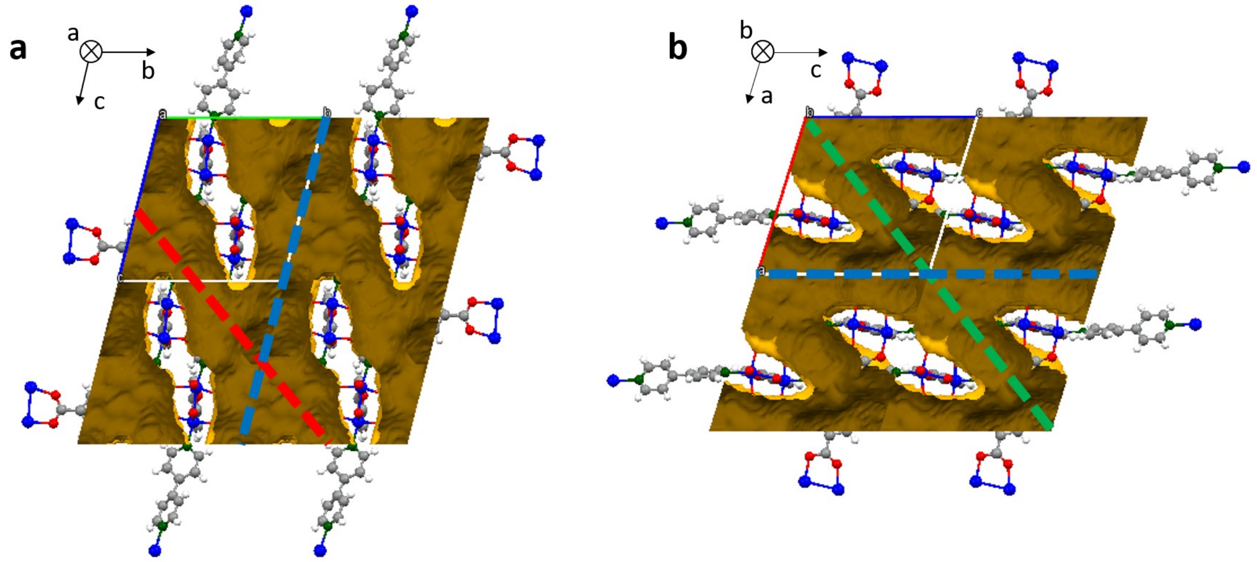

Supplementary Figure 15. Crystal structure of **2** including PEG. Void contour surface of **2** open by PEG viewed along the *a*-axis (**a**) and the *b*-axis (**b**). The PEG guest was removed using the SQUEEZE procedure. Nanochannels were present along three directions: [001], [101], and [011]. Electron density originating from the PEG guest was detected in the three types of nanochannels. Unidirectional pores are highlighted with dashed lines (blue: [001]; red: [011], green: [101]). Cobalt, carbon, nitrogen, oxygen, and hydrogen atoms are represented in blue, gray, green, red, and white, respectively.

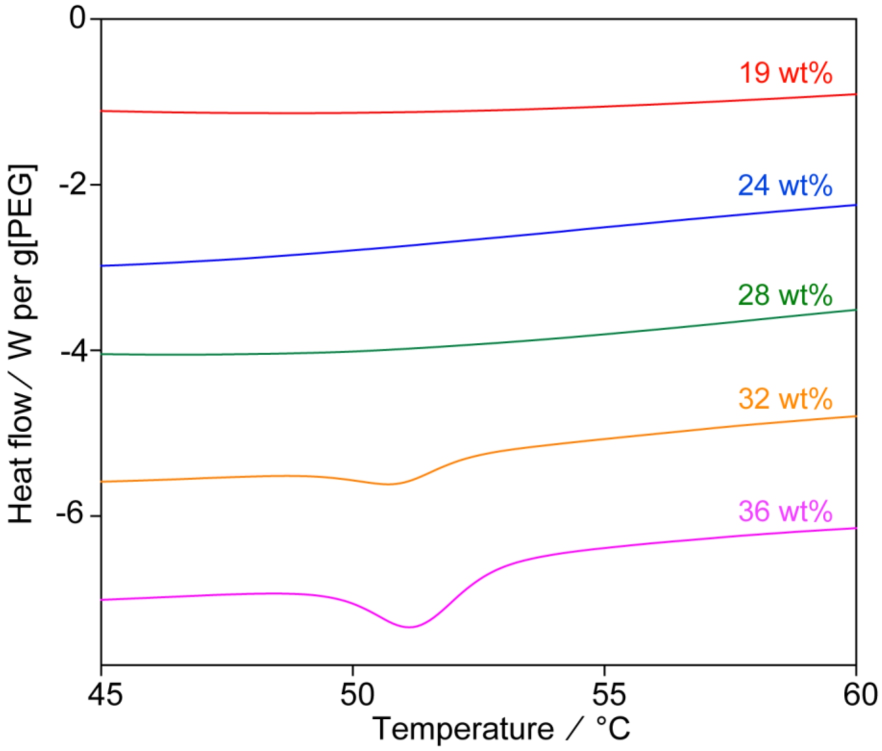

Supplementary Figure 16. DSC heating curves of **2** and H–PEG–H nanocomposites with various incorporation ratios of PEG prepared by the solvent-evacuation method. DSC was used to detect the endothermic peak at 52 °C corresponding to the melting of H–PEG–H outside of **2**. This peak appeared only if the amount of PEG is superior to the maximal capacity of the MOF, and corresponded to the excess PEG that could not fit in the channels of **2**. The maximal capacity was thus comprised between 28 and 32 wt% (reported to the mass of empty **2**). The traces correspond to the first cycle of the analysis, and samples were not thermally annealed before the measurement. M_n of PEG used in these experiments was 2 kDa. Scan rate: 10 °C min^{-1}.

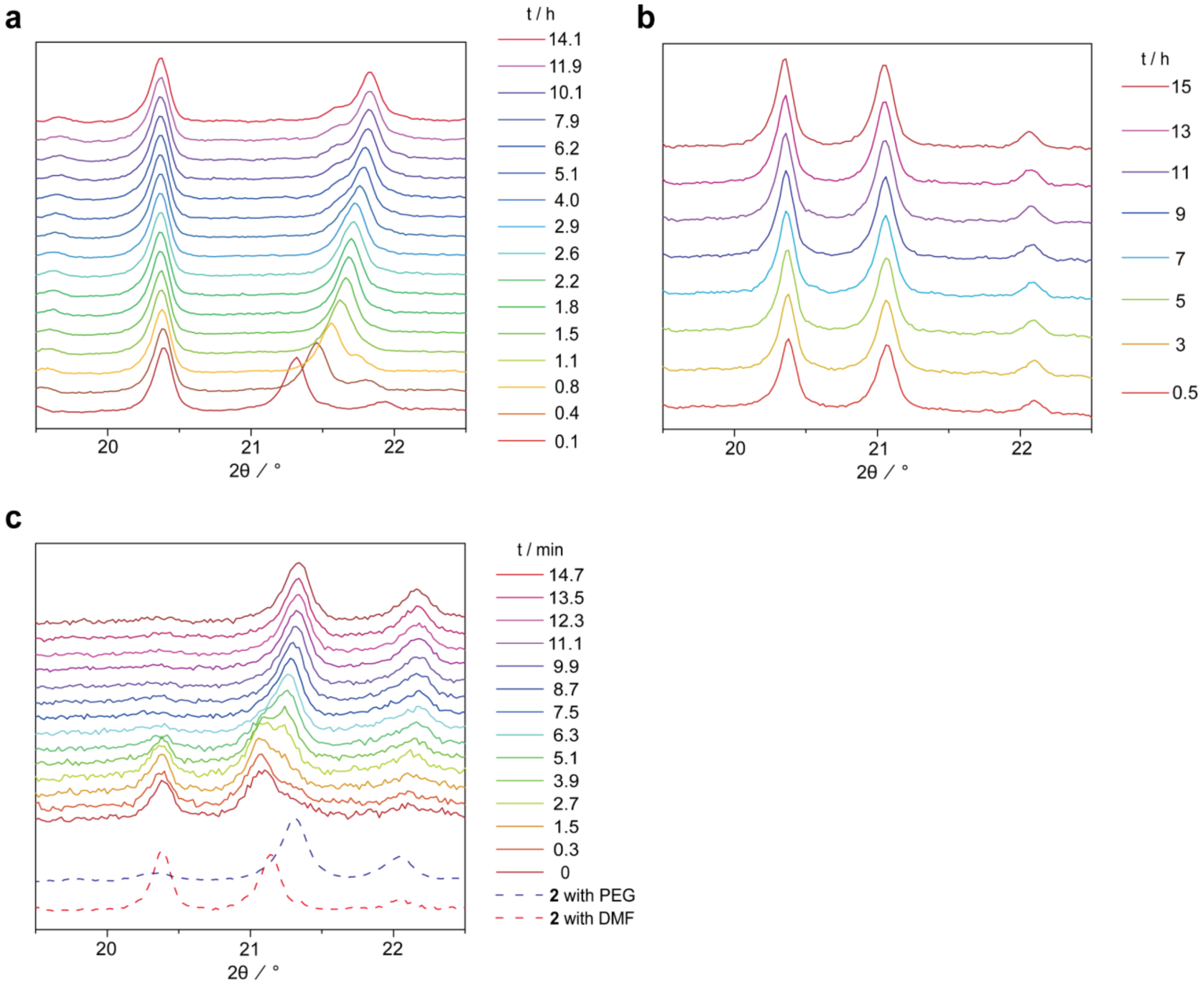

Supplementary Figure 17. Insertion of PEG in **2** upon solvent removal, monitored by *in situ* PXRD. (**a**) Time-resolved PXRD of **2** immersed in DMF, then evacuated under reduced pressure. (**b**) Time-resolved PXRD of **2** immersed in DMF solution of H–PEG–H. (**c**) Time-resolved PXRD of **2** immersed in DMF solution of H–PEG–H, then evacuated under reduced pressure. M_n of PEG used in these works was 2 kDa. In this study, DMF was used as a solvent because of characteristic peaks of **2** containing PEG and DMF, so that the individual phases can be clearly identified. Supplementary Fig. 17a presents the evolution of **2** with DMF under reduced pressure (<0.3 kPa). The initial phase has a composition of **2** and DMF. As DMF is evacuated, the peak at 21.1° is progressively shifted toward the larger angles and reaches its final position at 21.8° after 6 h. This peak does not move even after additional 8 hours in vacuum, revealing the existence of a stable adduct of **2** and DMF. This continuous shift of a unit cell in a flexible MOF is analogous to a recent report by Carrington *et al.*.[11] Interestingly, the peak at 20.4° remains unchanged upon DMF evacuation, and can be thus considered as a marker of the phase of **2**-DMF composite. Supplementary Fig. 17b presents the evolution of **2** maintained in contact with a solution of H–PEG–H in DMF. Formation of **2** including PEG was not observed, showing that the PEG insertion proceeds only if solvent is removed. Supplementary Fig. 17c presents the evolution of **2** in contact with a solution of H–PEG–H in DMF, as vacuum (0.3 kPa) was applied. Initially, **2** adopts the configuration of the DMF adduct. However, this phase disappears completely within 10 min to be converted into **2** containing PEG. This indicates that the encapsulation of PEG is caused by the solvent removal. Furthermore, DMF evacuation is significantly faster in the presence of PEG, revealing that PEG can displace the solvent to cause its own insertion.

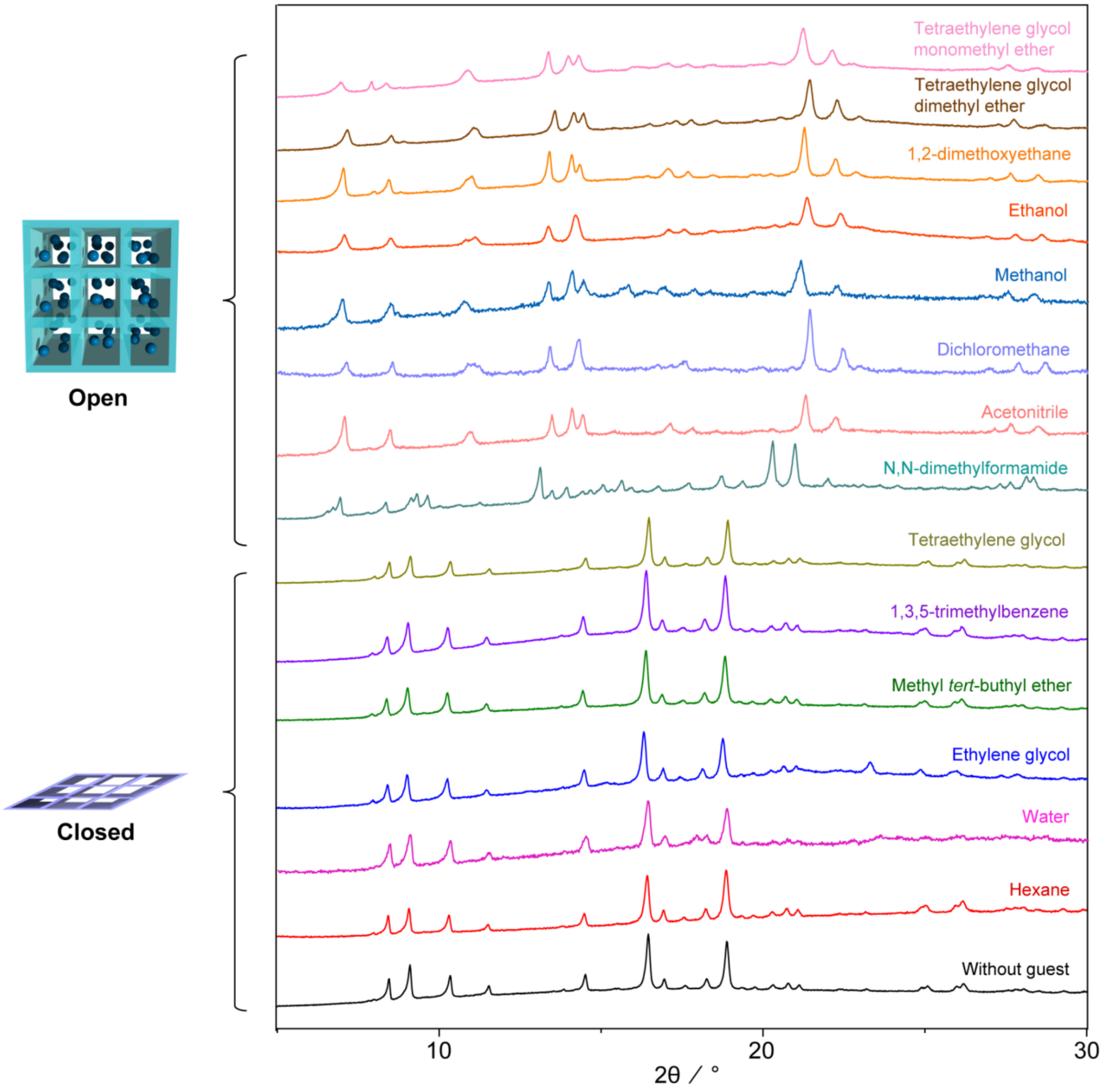

Supplementary Figure 18. PXRD patterns of **2** before and after immersion in various solvents. In apolar (e.g., hexane), highly polar (e.g., H_2O, ethylene glycol), or bulky solvent (e.g., 1,3,5-trimethylbenzene, methyl *tert*-butyl ether), **2** presents the pattern of a closed-pore phase, denoting the absence of insertion. In contrast, **2** presents an opened-pore phase pattern with diffraction peaks below 8° when immersed in solvents of intermediate polarity and small size, such as ethanol, acetonitrile, dichloromethane, and 1,2-dimethoxyethane. As observed for PEG of higher molecular weight, **2** presents a closed-pore phase pattern in tetraethylene glycol, but an open-pore phase pattern in tetraethylene glycol monomethyl ether and dimethyl ether.

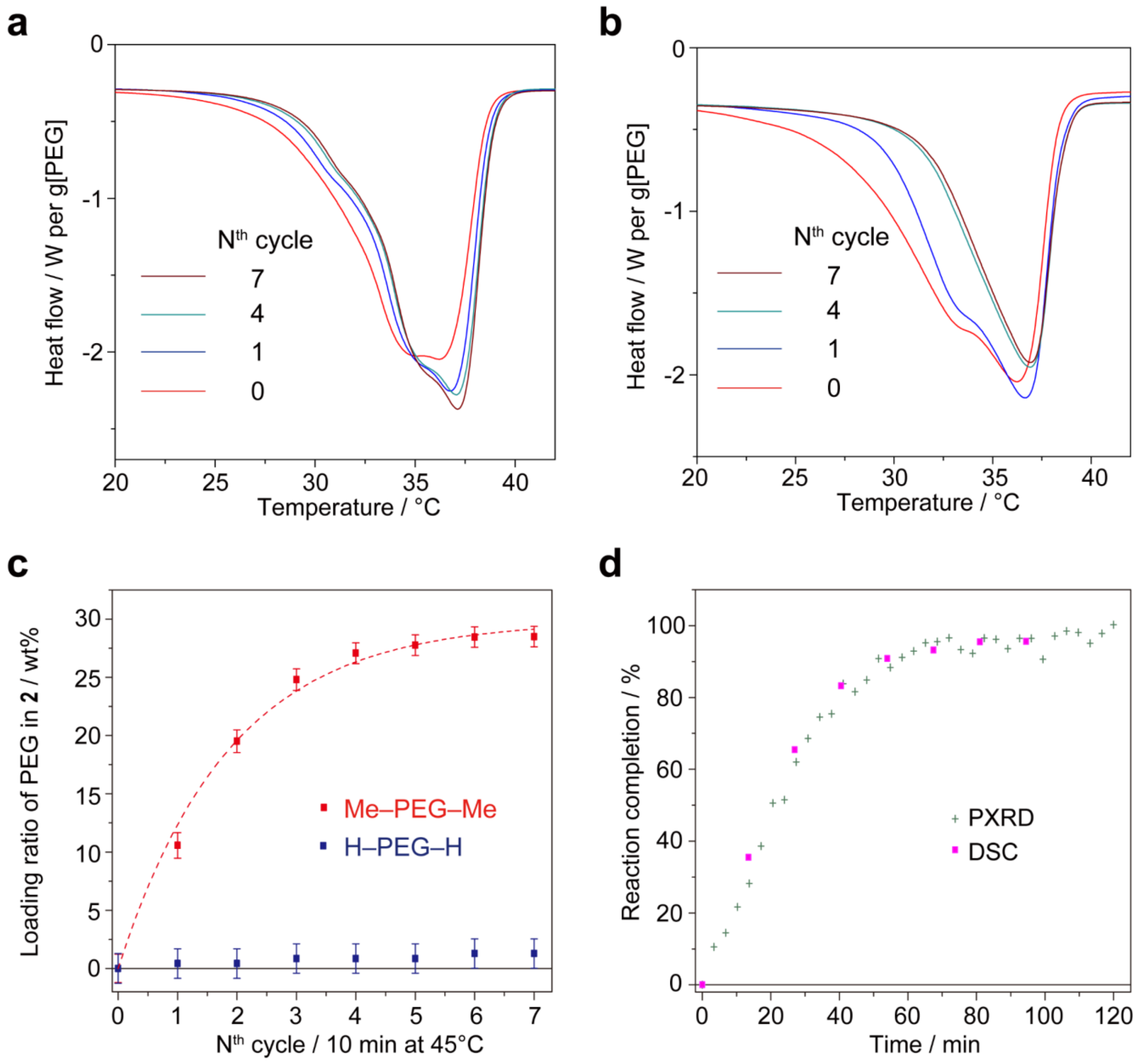

Supplementary Figure 19. Determination by DSC of the insertion kinetics of PEG in **2**. (**a**, **b**) DSC trace of H–PEG–H (**a**) and Me–PEG–Me (**b**) in contact with **2**. Scan rate was 5 °C min^{-1}. (**c**) Evolution of the loading *L* of PEGs into **2**, deduced from the DSC analysis. The loading of Me–PEG–Me was fitted by a single exponential $L(N) = L_{max}(1-exp(-N/N_0))$, indicating an apparent first order kinetics. The maximal loading L_{max} was 29.8 ± 0.6 w%. Note that in this case, the activity of molten PEG is always equal to 1, as it is a neat pure liquid. Dotted line: fit curve. Error bars: s.d. of the propagated measurement uncertainties. (**d**) Comparison of the insertion kinetics of Me–PEG–Me in **2**, obtained by DSC and PXRD analyses. Note that for the DSC analysis, the contact time of **2** with molten PEG was ca. 13 min per cycle (to account for the temperature increases and decrease after melting and before freezing respectively). The insertion kinetics of Me–PEG–Me observed by DSC (that monitors the insertion of PEG) is fully consistent with that observed by PXRD (where the disappearance of the closed-pore form of **2** was observed). M_n of PEG used in these experiments was 1 kDa.

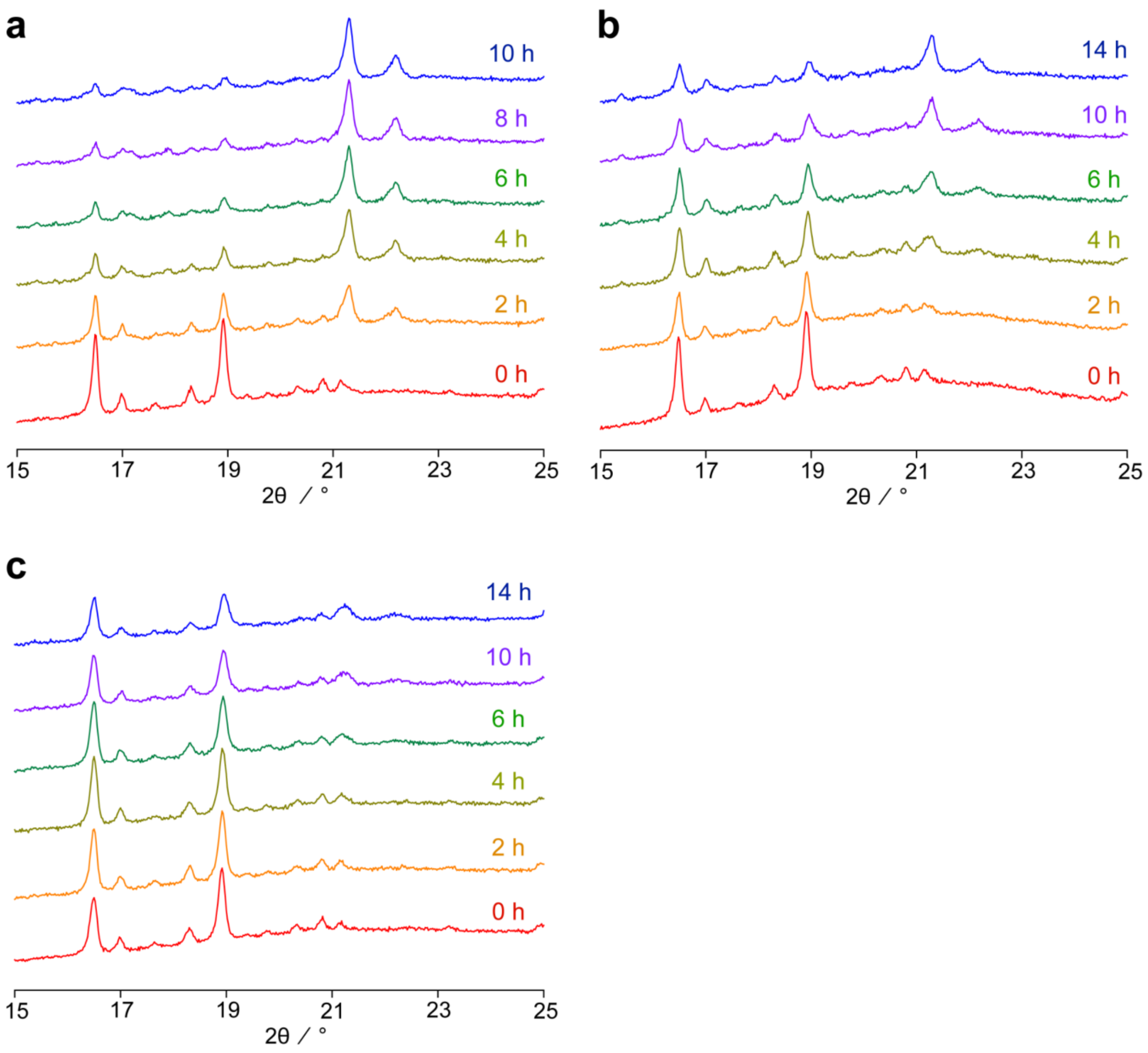

Supplementary Figure 20. Structural analysis of **2** upon PEG inclusion. Time-resolved in situ PXRD patterns of **2** in presence of (**a**) Et–PEG–Et, (**b**) Bu–PEG–Bu, and (**c**) H–PEG–Me recorded at 45 °C. M_n of PEG was 1 kDa. The closed-pore form of **2** is progressively converted into a phase opened by PEG. The kinetics of conversion is highly dependent on the nature of both terminal groups.

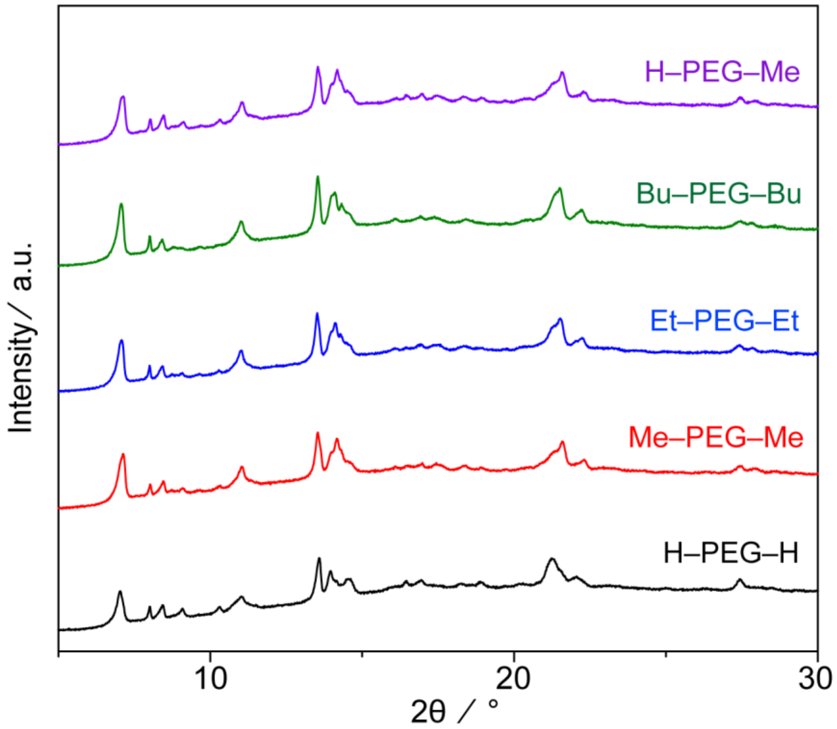

Supplementary Figure 21. PXRD patterns of **2** including H–PEG–H, Me–PEG–Me, Et–PEG–Et, Bu–PEG–Bu, and H–PEG–Me obtained by the solvent-evacuation method at room temperature. With this method, encapsulation of all R^1–PEG–R^2 (with R^1; R^2:H, Me, Et, and Bu) could be achieved readily. This is in sharp contrast with the direct introduction of molten PEG that did not proceed for H–PEG–H. M_n of PEG used in these experiments was 1 kDa.

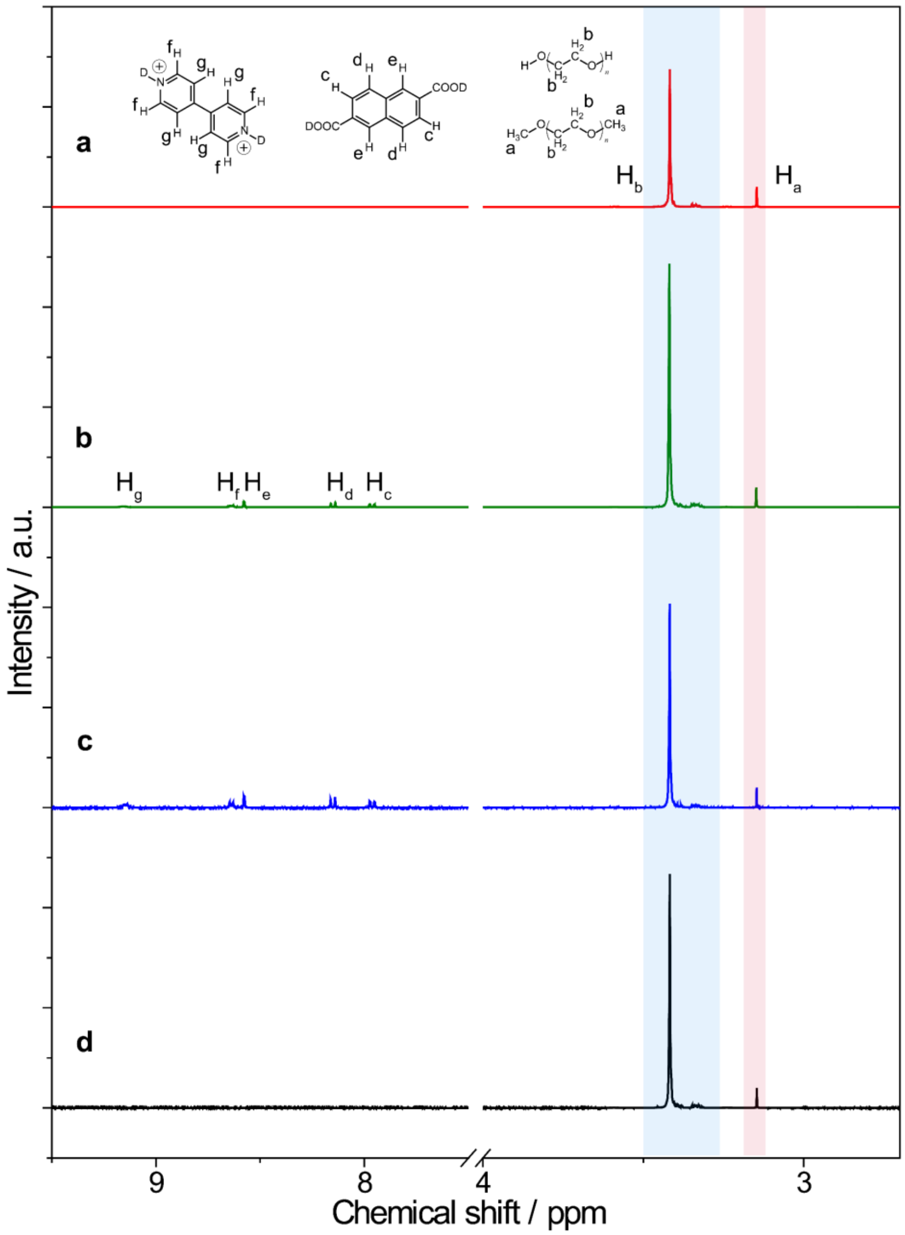

Supplementary Figure 22. Purity of PEG after the separation using **2**. ^{1}H-NMR spectra of (**a**) pure Me–PEG–Me; (**b**–**c**) composite of **2** and a mixture of Me–PEG–Me and H–PEG–H after insertion, before (**b**) and after (**c**) removal of excess PEG by blotting. (**d**) PEG collected outside of **2**, extracted from the blotting paper. Spectra were collected in a mixture (9:1 v/v) of D_3CSOCD_3 and DCl (37 wt% in D_2O). The separation was accomplished by putting empty **2** in contact with a mixture of Me–PEG–Me and H–PEG–H, in large excess (H–PEG–H and Me–PEG–Me correspond to 100 wt% and 100 wt%, respectively). The initial integral of I_{EG} was equal to 188.7, for a total PEG loading of L_{Initial} = 200 wt%. After insertion, PEG outside of **2** was removed as far as possible by blotting with filter paper. The resulting composite had thus a loading L_{Blotted} of 75 wt%, with an integral $I_{\mathrm{EG,Blotted}}$ of 161.7. This corresponds to an overall enrichment in Me–PEG–Me, with a selectivity of 1.4. This value takes into account the presence of PEG inside **2**, rich in Me–PEG–Me, and residual PEG outside **2**, enriched in H–PEG–H ($I_{\mathrm{EG,Outside}} = 194.5$), with $L_{\mathrm{Blotted}} = L_{\mathrm{Inside}} + L_{\mathrm{Outside}}$. By assuming a value for L_{Inside}, the integral for EG inside **2** ($I_{\mathrm{EG,Inside}}$) can be determined with the formula $L_{\mathrm{Blotted}} * I_{\mathrm{EG,Blotted}} = L_{\mathrm{Inside}} * I_{\mathrm{EG,Inside}} + L_{\mathrm{Outside}} * I_{\mathrm{EG,Outside}}$. Considering the maximal loading ($L_{\mathrm{Inside,Max}}$ = 29.8 wt%) as an upper bound, the selectivity for the insertion of Me−PEG−Me into **2** was estimated to be at least of 5.2.

Supplementary Table 1. Crystal data and structure refinement for **2** including H−PEG−H (0.6 kDa) obtained by the solvent-evacuation method.[a]

**Formula**	$C_{34} H_{20} Co_2 N_2 O_8$
**Formula weight**	702.38
**Temperature (K)**	103(2)
**Wavelength (Å)**	0.71073
**Crystal system**	triclinic
**Space group**	$P\bar{1}$
a **(Å)**	13.1456(12)
b **(Å)**	13.1962(13)
c **(Å)**	13.8838(12)
α	101.838(8)
β	105.372(8)
γ	96.160(8)
V **(Å^3)**	2239.3(4)
Z	2
**Calcd Density (g cm^{-3})**	1.042
μ **(mm^{-1})**	0.779
F**(000)**	712
**Crystal size (mm^3)**	0.05 × 0.03 × 0.01
**Total reflection**	46158
**Unique reflection**	13234
R_{int}	0.2156
**Goodness-of-fit**	0.934
**Final R_1 and wR_2 indices [$I > 2\sigma(I)$]**[b]	0.0835, 0.1968
**R_1 and wR_2 indices (all data)**[c]	0.2351, 0.2480
**max, min $\Delta\rho$ (eÅ^{-3})**	0.798, -0.841
**CCDC number**	1836242

[a] Data based on the PLATON/SQUEEZE[3,4] model. [b] $R_1 = R = \Sigma \,||F_o| - |F_c|| / \Sigma |F_o|$. [c] $wR_2 = [\Sigma w(F_o^2 - F_c^2)^2 / \Sigma w(F_o^2)^2]^{1/2}$.

Supplementary Table 2. ^{1}H-NMR peak integrals for terminal methyl (I_{Me}), main chain ethylene glycol (I_{EG}) and 2,6-naphthalenedicarboxylic acid (I_{NDC}, protons in position 3 and 7).

Sample	I_{Me} (H_a)	I_{EG} (H_b)	I_{NDC} (H_c)
Pure Me−PEG−Me	6	93.3	N/A
Composite before blotting	6	188.7	5.9
Composite after blotting	6	161.7	13.5
PEG outside **2**	6	194.5	N/A

Supplementary References

1. O. V. Dolomanov *et al.*, OLEX2: A complete structure solution, refinement and analysis program. *J. Appl. Cryst.* **42**, 339-341 (2009).
2. G. M. Sheldrick, SHELXT - Integrated space-group and crystal-structure determination. *Acta Cryst. C* **71**, 3-8 (2015).
3. A. L. Spek, PLATON 99, A Multipurpose Crystallographic Tool (Utrecht University, Utrecht, The Netherlands, 1999).
4. A. L. Spek, PLATON SQUEEZE: a tool for the calculation of the disordered solvent contribution to the calculated structure factors. *Acta Cryst. C* **71**, 9-18 (2015).
5. T. Uemura *et al.*, Molecular-Level Studies on Dynamic Behavior of Oligomeric Chain Molecules in Porous Coordination Polymers. *J. Phys. Chem. C* **119**, 21504-21514 (2015).
6. Chun, H., Dybtsev, D.N., Kim, H. & Kim, K. Synthesis, X-ray Crystal Structures, and Gas Sorption Properties of Pillared Square Grid Nets Based on Paddle-Wheel Motifs: Implications for Hydrogen Storage in Porous Materials. *Chem. Eur. J.* **11**, 3521-3529 (2005).
7. Dybtsev, D.N., Chun, H. & Kim, K. Rigid and Flexible: A Highly Porous Metal–Organic Framework with Unusual Guest-Dependent Dynamic Behavior. *Angew. Chem. Int. Ed.* **43**, 5033-5036 (2004).
8. Aggarwal, H., Das, R.K., Bhatt, P.M. & Barbour, L.J. Isolation of a structural intermediate during switching of degree of interpenetration in a metal-organic framework. *Chem. Sci.* **6**, 4986-4992 (2015).
9. Jaoued-Grayaa, N., Boughariou-Charrada, B. & Hedhli, A. Synthesis and the Structure to Property Relationship of Monoperfluoroalkyl Polyethylene Glycol. *J. Surfactants Deterg.* **17**, 767-772 (2013).
10. Gentilini, C., Boccalon, M. & Pasquato, L. Straightforward Synthesis of Fluorinated Amphiphilic Thiols. *Eur. J. Org. Chem.* **2008**, 3308-3313 (2008).
11. E. J. Carrington *et al.*, Solvent-switchable continuous-breathing behaviour in a diamondoid metal-organic framework and its influence on CO_2 versus CH_4 selectivity. *Nature Chem.* **9**, 882-889 (2017).
